# Rapid Detection of β-Lactamase-Producing Bacteria Using the Integrated Comprehensive Droplet Digital Detection (IC 3D) System

**DOI:** 10.3390/s20174667

**Published:** 2020-08-19

**Authors:** Yiyan Li, Hemanth Cherukury, Louai Labanieh, Weian Zhao, Dong-Ku Kang

**Affiliations:** 1 Department of Physics and Engineering, Fort Lewis College, Durango, CO 81301, USA; yli@fortlewis.edu; 2 Department of Pharmaceutical Sciences, University of California Irvine, Irvine, CA 92697, USA; hcheruku@uci.edu (H.C.); weianz@uci.edu (W.Z.); 3 Sue and Bill Gross Stem Cell Research Center, University of California Irvine, Irvine, CA 92697, USA; 4 Department of Bioengineering, Stanford University, Stanford, CA 94305, USA; llabanie@stanford.edu; 5 Chao Family Comprehensive Cancer Center, University of California Irvine, Irvine, CA 92697, USA; 6 Edwards Life Sciences Center for Advanced Cardiovascular Technology, University of California Irvine, Irvine, CA 92697, USA; 7 Department of Biomedical Engineering, University of California Irvine, Irvine, CA 92697, USA; 8 Department of Biological Chemistry, University of California Irvine, Irvine, CA 92697, USA; 9 Department of Chemistry, Incheon National University, Incheon 22012, Korea; 10 Research Institute of Basic Sciences, Incheon National University, Incheon 22012, Korea

**Keywords:** IC 3D, droplet microfluidics, digital quantification

## Abstract

Antibiotic-resistant bacteria have emerged as an imminent global threat. The lack of rapid and sensitive diagnostic techniques leaves health care providers with inadequate resources for guiding therapy and risks the lives of patients. The traditional plate culturing methods for identifying antibiotic-resistant bacteria is laborious and time-consuming. Bulk PCR (Polymerase Chain Reaction) and qPCR are limited by poor detection sensitivity, which is critical for the early-stage detection of bloodstream infections. In this study, we introduce a technique for detecting β-lactamase-producing bacteria at single-cell sensitivity based on a commercial β-lactamase sensor (Fluorocillin), droplet microfluidics, and a custom 3D particle counter. Bacteria-containing samples were encapsulated within picoliter-sized droplets at the single-cell level and cultured within water-in-oil droplets containing antibiotics and the Fluorocillin sensor. Then, fluorescent droplets were digitally quantified with the 3D particle counter, which is capable of analyzing milliliter-scale volumes of collected droplets within 10 min. The fluorescence signal from single-colony droplets was detectable in less than 5 h, and the 3D scanning was performed in less than 10 min, which was significantly faster than conventional culture-based methods. In this approach, the limit of detection achieved was about 10 bacterial cells per mL of sample, and the turnaround time from sample to result was less than 6 h. This study demonstrates a promising strategy for the detection of β-lactamase-producing bacteria using the recently developed IC 3D system.

## 1. Introduction

Due to the use of β-lactam antibiotics in medicine, agriculture, and veterinary medicine, bacteria have evolved to survive in the presence of antibiotics by mutating and encoding extended-spectrum β-lactamases (ESBLs). The evolution of ESBLs has been faster than the cycle of developing new drugs, which leads to a high failure rate of treating infections caused by ESBLs. The situation is especially dire when bacteria enter the bloodstream and cause bloodstream infections (BSI). Without proper treatment, BSI can lead to sepsis and result in a very high mortality rate. An estimated 20% of all annual deaths are caused by complications due to sepsis, making up nearly 50% of all deaths in the hospital [1]. Nearly 1 million individuals are hospitalized due to sepsis annually in the US alone with at least 30,000 of those patients dying in the hospital, and the numbers are rising every year [2]. The high mortality rate of sepsis can be attributed to the lack of rapid diagnostics tools to direct the proper administration of antibiotics by healthcare providers. The current gold standard for bacterial infection diagnosis remains the 100-year-old plate culture method, which can take up to 5 days to grow the pathogen to detectable levels, and even longer for confirmation of a negative result [3]. Until the results of the culture are confirmed, physicians are generally directed to administer a cocktail of broad-spectrum antibiotics in the hopes that one or more of the antibiotics will be effective in curbing the infection [4,5]. However, this empirical treatment is ineffective in up to 50% of patients [6]. Compounding the issue is the high incidence of drug-resistant bacteria and especially multi-drug resistant (MDR) bacteria, the prevalence of which is further increased by nonspecific broad-spectrum antibiotic administration [7,8]. Therefore, there is an urgent need for a rapid diagnostic test that can give sample-to-result in less than 24 h.

More recently, nano- and micro-based systems have been developed such as Droplet Digital PCR (ddPCR) [9,10,11], which involves the partitioning of the sample into thousands or millions of droplets, followed by separate PCR reactions occurring within each individual droplet. The number of DNA copies contained in each droplet can be predicted by the Poisson distribution [12]. Samples with low target concentrations can be partitioned into millions of nanoliter-sized droplets with a low probability that any droplet will contain more than one target. Once the reaction is complete, the positive droplets will fluoresce, which allows for counting by a flow cytometer to determine the number of positive droplets with single-digit sensitivity. Compared to traditional qPCR, ddPCR can improve detection sensitivity and selectivity but typically is limited to small sample volume (μLs), which cannot handle the required clinical sample volume (mLs of blood) and throughput, as bacteria in patient samples may be present at single-digit CFU levels. These conventional methods tend to require expensive equipment and complex sample processing steps (e.g., cell lysis, nucleic acid extraction, amplification of signal) to purify and enrich the target, resulting in significant target loss when there are only a low number of targets present in the sample [13].

The majority of molecular diagnostic assays for antibiotic resistance have targeted genetic markers; however, these do not always correlate to the observed phenotype. On the other hand, phenotypic assays such as antibiotic susceptibility testing and β-lactamase activity assays act as a more direct functional readout for antibiotic resistance [14,15]. β-lactams are historically the most successful antibiotics and account for the majority of antibiotics prescribed today [16]. They work by inhibiting the synthesis of the bacterial cell wall. Resistance to β-lactams is primarily mediated through enzymes, known as β-lactamases, which hydrolyze the β-lactam ring of these antibiotics, rendering them ineffective [17]. Common pathogenic Enterobacteriaceae such as *K. pneumoniae* and *E. coli* typically carry extended spectrum β-lactamase (ESBL) or carbapenem resistance (CRE), and they are among the most prevalent antimicrobial-resistant pathogens [18,19]. ESBLs and CREs account for 10–20% of serious *E. coli* infections and 20–30% of *Klebsiella* infections reported nationally [20]. ESBLs mediate resistance to extended-spectrum β-lactam antibiotics, including cephalosporins and monobactams. Even worse are the carbapenemases produced by CREs that can hydrolyze almost all β-lactam antibiotics including carbapenems, which are typically used as the “drug of last resort” to treat severe infections by Gram-negative bacteria [21]. Indeed, the mortality rate of bloodstream infections (BSI) due to CREs is over 50% [22].

**Figure 1:**
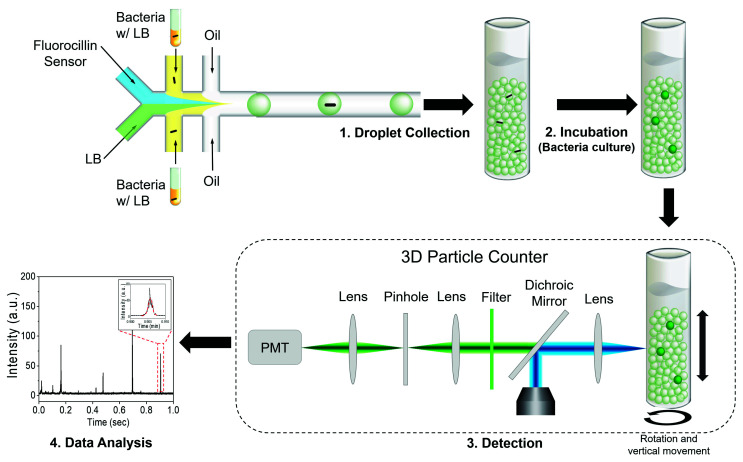
The diagram of the single-bacterium detection using the Integrated Comprehensive Droplet Digital Detection (IC 3D) system. The presence of a single bacterium in a microfluidic droplet results in detectable fluorescence when excited by a laser. The IC 3D droplet counter quantifies the positive spikes and reports the bacteria counts in the sample.

We recently reported a technology termed Integrated Comprehensive Droplet Digital Detection (IC 3D), which is capable of analyzing milliliters of droplets within minutes to identify target pathogens (Figure 1) [23,24,25,26].

Here, we report a phenotypic droplet assay for β-lactamase-mediated antibiotic resistance. Basically, bacterial cells were encapsulated within 60 μm droplets with antibiotics and a fluorescence-producing β-lactamase sensor. After encapsulation, only antibiotic-resistant bacteria can be grown inside droplets, and fluorescent droplets indicate β-lactamase production from grown bacteria. This is the first report of the detection and quantification of β-lactamase-producing bacteria in a relatively large volume of sample (1 mL) without sample preparation and pre-cultivation using droplet-based microfluidics. In this study, clinically relevant samples were rapidly analyzed in a highly sensitive manner by integrating phenotypic assay with IC 3D technology. This phenotypic approach for the monitoring of antibiotic-resistant bacteria in droplets avoids the pitfalls and expense of molecular-based assays while being significantly faster than traditional culture-based methods.

## 2. Materials and Methods

### 2.1. Bacteria Preparation

The STAR and AD494 strains are engineered ampicillin-resistant *Escherichia coli* (*E. coli*) and were provided by Dr. Jianghong Rao (Stanford University). Ampicillin-susceptible Top10 *E. coli* (Thermo Fisher, Waltham, MA, USA) and *Bacillus subtilis* (*B. subtilis*, ATCC^®^ 82™, ATCC, Manassas, VA, USA) were purchased and the other ampicillin-susceptible K12 *E. coli* was a kind gift from Dr. Manuela Raffatellu (UC Irvine). De-identified clinically isolated bacteria (#41 and #47) were obtained from Dr. Ellena Peterson through the UC Irvine Pathology Research Biorepository. A single colony, freshly grown on an agar plate containing lysogeny broth (LB), was inoculated into 5 mL of LB and incubated with shaking at 37 °C for 14 h. Ten (10) μL of this culture was further inoculated in 5 mL fresh LB for 4 h, and the concentration of bacterial cells was determined by the colony-forming unit (CFU) assay using freshly cultured bacterial cells. *E. coli* K12 or other *E. coli* strains were freshly cultured as described above. Then, two mL of cultured cells were serially diluted in 10-fold intervals with LB and divided into two groups: one for stock with 20% glycerol and the other for the CFU assay. Cell concentrations ranging from the 4th to the 9th dilution were then plated onto the agar-LB plates using 100 μL of diluted cells. Then, the bacterial suspension was spread evenly over the entire surface of the plate. After drying, the plates were taped to prevent contamination and incubated at 37 °C overnight or until colonies formed. After the colonies reached a suitable size, they were counted to determine the cell concentration.

### 2.2. Synthesis of PFPE–PEG–PFPE Surfactant

The biocompatible fluorinated surfactant used for droplet generation was synthesized as previously described by Chen et al. [27]. Krytox 157FS (H) (50 g, MW: ~5000 g/mol, Dupont, Londonderry, UK) was dissolved in 50 mL anhydrous HFE-7500 with excess oxalyl chloride (12.5 g, Sigma-Aldrich, Gillingham, UK) and stirred overnight at 85 °C under argon. The solvent was removed through rotary evaporation and high vacuum. The resulting light-yellow product was mixed with Jeffamine XTJ 501 (3.5 g, MW: 900 g/mol, Sigma-Aldrich, UK) and dissolved in a mixture of 50 mL HFE-7500 with 50 mL of anhydrous dichloromethane (50 mL, Sigma-Aldrich, UK) at 65 °C with stirring for 2 days under an argon atmosphere, resulting in a milky white product after rotary evaporation. Insoluble white particles were removed through centrifugation at 8000 rpm for approximately 10 min and dried using a vacuum desiccator for 24 h. Then, the surfactant was used without any further purification.

### 2.3. Fabrication of Droplet-Based Microfluidic Chip and Droplet Generation

The pattern of the microchannel (width: 10–200 µm, depth: 20–50 µm) was designed in AutoCAD and printed on high-resolution film masks by CAD/Art Services (Bandon, OR, USA). Two aqueous inlets are used to deliver the Fluorocillin sensors and the bacteria sample to the microfluidic channels separately to minimize the reaction before the droplet formation. The channel design was transferred to 4-inch silicon wafers by the standard photolithography procedure, which follows. The wafers were briefly rinsed with 5% hydrofluoric acid (Sigma-Aldrich, St. Louis, MO, USA) and deionized (DI) water. Before spin coating (6NPP-LITE, Laurell Technologies Corporation, North Wales, PA, USA), wafers were dehydrated in an oven at 95 °C for 10 min. Then, negative photoresist (approximately 3 g, SU-8 50, MicroChem, Chestech, UK) was spin-coated (500 rpm for 10 s, then 3000 rpm for 30 s) onto the wafer. Then, the SU-8 layer was cured on a hotplate at 65 °C for 5 min and at 95 °C for 30 min. Then, the cured SU-8 layer was exposed to UV radiation (14 s, 20 mW/cm^2^, AB&M INC UV Flood Exposure System) through the photomask, and the wafer was subsequently post-baked for 1 min (65 °C) and 5 min (95 °C). Unexposed SU-8 was removed by soaking in SU-8 developer for 5 min. Then, the wafer was cleaned using isopropyl alcohol, blow-dried with filtered nitrogen gas, and silanized with perfluorooctyl-trichlorosilane (Sigma-Aldrich, St. Louis, MO, USA) under vacuum for 3 h.

PDMS (Polydimethylsiloxane, Sylgard 184, Dow Corning, Midland, MI, USA) and its curing agent were mixed in the ratio of 10:1 *w*/*w*. The mixture was stirred thoroughly to avoid any uncured PDMS base and followed by a 20-min degassing process in a vacuum chamber. Then, the mixture was poured onto the patterned silicon wafer mold and fully cured in an oven (at least 4 h at 65 °C). The cured PDMS pieces were peeled off from the wafer and bonded to cleaned glass slides following the plasma treatment.

**Figure 2:**
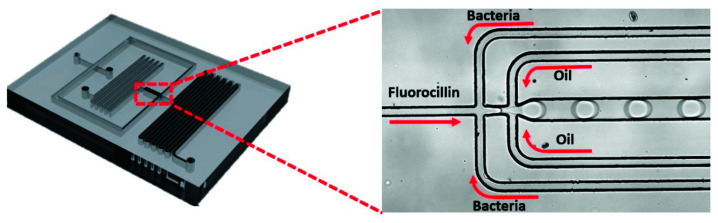
Encapsulation of β-lactamase-producing bacteria into microdroplets using microfluidic chips.

Various concentrations of *E. coli* K12 or other strains of bacterial cells were prepared through serial dilution and subsequently spiked in LB. Then, bacterial cell-spiked LB samples were microencapsulated with 2 µM Fluorocillin sensor using a droplet microfluidic device. Droplets were collected within 2 mL cuvettes for incubation at 3 °C without rotation for 5 h (Figure 2).

The engineering oil (HFE-7500) was mixed with the homemade polyethyleneglycol surfactant (1.8% *w*/*w*) and used as the continuous oil phase for microfluidic droplet generation. HFE-7500 has a low viscosity (0.77 at 25 °C), a high boiling point (128 °C), and a high density (1614 kg/m^3^), which makes it a good candidate for generating microfluidic droplets. The surfactant used in this study has been used with the HFE-7500 oil to stabilize the emulsion and prevent the droplets from coalescing even at 100 °C. The oil and the aqueous phase (Fluorocillin sensor and bacteria culture) were injected into the microfluidic device through syringes and tubing by the PHD 2000 syringe pumps (Harvard Apparatus). The flow rate was limited in the 0.5 to 5 μL min^−1^ range.

### 2.4. Identification of β-Lactamase Production in Bulk

**Figure 3:**
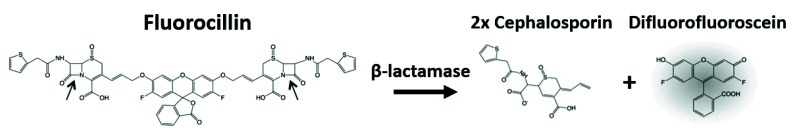
The detection mechanism of bacterial β-lactamase using Fluorocillin.

The fluorescence-based analysis of β-lactamase production with Fluorocillin sensor (Fluorocillin™ Green β-Lactamase Substrates, Invitrogen, Carlsbad, CA, USA), as shown in Figure 3, was performed using a fluorescence microplate reader (Synergy HT, Biotek, Winooski, VT, USA) with an excitation wavelength of 488 nm and an emission wavelength of 520 nm. To initiate the reaction in the microplate reader, 5 μL of a concentration stored-stock sample (1 × 10^9^ or different number of bacterial cells) and 25 μL of 2× LB were mixed in the microwell plate and 10 μL of H_2_O was added to adjust the total volume up to 50 μL.

Microwell plates were placed into a microplate reader and the fluorescence intensity was monitored at 3 min intervals continuously following the addition of 10 μL of 20 μM Fluorocilliin sensor into each well. The fluorescence intensity was recorded for 120 min. All the experiments were performed in triplicate.

### 2.5. Fluorescence Detection Using the IC 3D System

The 3D particle counter is the core instrument in the IC 3D system (Figure 1). The 3D particle counter was developed by Dr. Gratton and colleagues at UC-Irvine. The particle counter consists of a narrow beam laser and a dichroic mirror to reflect the laser beam to the sample through an objective lens. The fluorescence emission from the sample is collected by the same objective lens and passes the dichroic mirror, a bandpass filter, and finally reaches the PMT (Photomultiplier tube). The PMT outputs analog electrical signals to a trans-impedance amplifier (TIA), which is followed by a digitizer within the instrument’s box. The digital signal is sent to a desktop through a USB cable for data analysis. The 3D particle counter can robustly and accurately detect single-fluorescent particles from milliliter volumes within minutes [28,29].

Droplets containing bacterial cells in LB and 2 µM Fluorocillin sensors were collected in a cuvette following droplet generation, and droplets were incubated at 37 °C for 5 h for the cultivation. Then, 100 μL of the generated droplets were mixed with 1.9 mL of background droplets (pre-made LB droplets that contain 2 µM Fluorocillin without any bacteria) in a cleaned cuvette and followed by the 37 °C incubation. Following incubation, 2 mL of droplets were analyzed for 3–10 min in the 3D particle counter. The 3D particle counter was set to rotate at 400 rpm horizontally and move 10 mm s^−1^ vertically during the scanning operation. The time-domain fluorescence signals were collected by the PMT and converted to voltage signals for analysis. The collected voltage intensity waveforms were analyzed with a pattern-recognition filter in software (SimFCS, Laboratory for Fluorescence Dynamics, UC-Irvine, Irvine, CA, USA). The pattern-recognition software has an algorithm to differentiate the positive waveforms from noisy spikes based on the information of fluorescence intensity, cuvette rotation speed, and droplet size given by the users. The concentration of bacteria was determined by fitting the positive counts into a counts-concentration curve that was previously created using standard concentration samples.

## 3. Results

### 3.1. Detection of β-Lactamase-Producing Bacteria in a Bulk Assay

**Figure 4:**
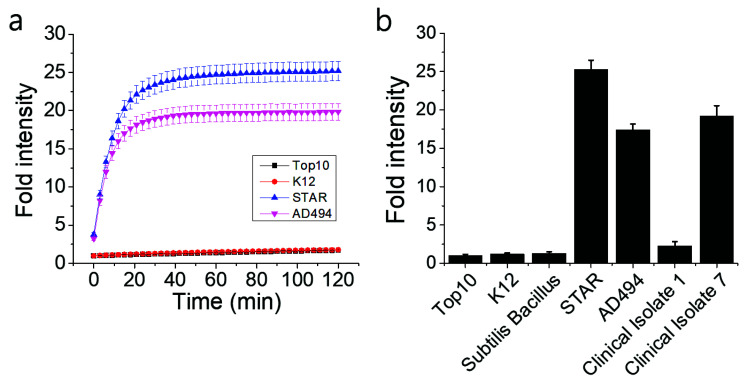
Fluorescence intensity collected by a 96-well microplate reader showed that the β-lactamase-producing bacteria were detected by the Fluorocillin sensor. (**a**) The STAR and the AD494 strains were *E. coli* engineered with ampicillin resistance, while K12 and Top10 were ampicillin-susceptible *E. coli*. (**b**) Fluorescence intensity of all the bacteria selected for this experiment.

The functionality of the Fluorocillin sensor was verified in terms of the detection of β-lactamase-production from bacteria with the 96-well microplate reader. Genetically engineered ampicillin-resistant *Escherichia coli* (*E. coli*), the STAR and the AD494 strains, produce β-lactamase and were used as a known positive control. The fluorescence intensity was generated from the β-lactamase-initiated cleavage of Fluorocillin, which was saturated for 30 min after both STAR and AD494 strains were exposed to the substrate (Figure 4a). In this study, two types of clinical isolates were also tested, and strong fluorescence intensity was observed with clinical isolate 7, which is the ampicillin-resistant strain (Figure 4b), but the ampicillin-susceptible strain (Clinical Isolate 1) showed very weak fluorescence intensity. None of the ampicillin-susceptible strains (Top10, K12, *Bacillus subtillis*, and Clinical Isolate 1) showed any strong fluorescence signals.

**Figure 5:**
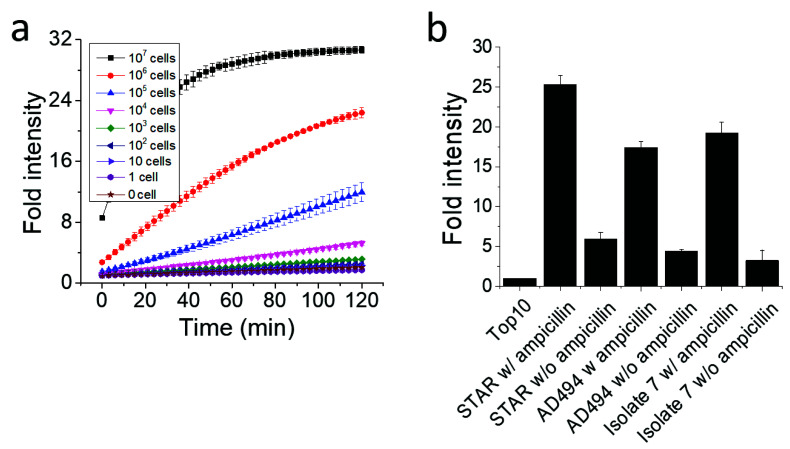
The effect of the bacteria concentration and the presence of ampicillin on the signal intensity. (**a**) The sensitivity of the Fluorocillin in the bulk assay (STAR strain). (**b**) Bacterial cells were cultured with or without ampicillin.

In this experiment, 1×10^9^ bacterial cells were loaded in each well of a 96-well plate in Figure 4, and the high number of bacterial cells produced enough β-lactamase that could reach a plateau of fluorescence intensity within 30 min. To verify the limit of detection for the β-lactamase-producing bacteria with Fluorocillin in a bulk assay, STAR strains were serially diluted, and fluorescence intensity was monitored for 120 min at room temperature (Figure 5). For every 10-fold concentration increase, the signal intensity and the response time showed significant differences (Figure 5a). The sample with the highest bacterial concentration showed the strongest signal intensity and saturated sooner. Consistent with the data in Figure 4, the 10^7^ samples showed a saturated fluorescence signal 30 min after the bacteria were exposed to the sensor. The sensitivity per well (limit of detection) was around 1 × 10^4^ cells per well.

The presence of ampicillin in the culture also had a positive impact on enhancing the signal intensity (Figure 5b). Three different bacteria strains were pre-cultured with or without ampicillin to investigate the difference of β-lactamase production. Each strain was loaded at a cell number of 1×10^7^, and fluorescence intensity was monitored after 120 min, with the Top10 ampicillin-susceptible strain serving as a negative control. As shown in Figure 5b, ampicillin co-culture increased β-lactamase production because it modulates the capacity of β-lactamase production, and fluorescence intensity was identified as 4.35, 4.04 and 5.71-fold higher in the STAR, AD494, and isolate 7 strains, respectively.

**Figure 6:**
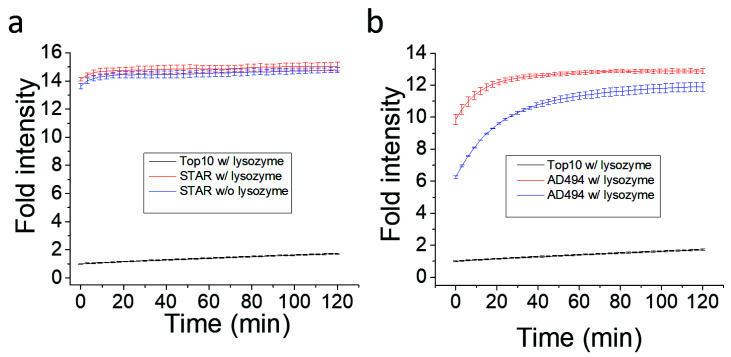
STAR cell line produce more/or active β-lactamase for Fluorocillin sensor. (a) β-lactamase activity in STAR strain (**a**) and AD494 strain (**b**).

β-lactamase is synthesized inside bacteria and secreted, which resulted in exposure to the Fluorocillin sensor. It was believed that bacterial lysis may induce or increase the sensitivity of β-lactamases detection with Fluorocillin. To investigate the effect of cell lysis, Fluorocillin and 1 × 10^6^ bacterial cells were incubated with or without lysozyme (Figure 6). Once bacterial cells were lysed with lysozyme, fluorescence intensity saturated more quickly compared to non-lysed cells, using the AD494 strain for the comparison. However, the STAR cell line only showed minor improvement, while the AD494 cell line showed aggressive improvements at the starting point of the experiment. This result suggests that the cell membrane of STAR stain may be more resistant against lysozyme.

### 3.2. Identification of β-Lactamase-Producing Bacteria with Droplet-Based Microfluidics

**Figure 7:**
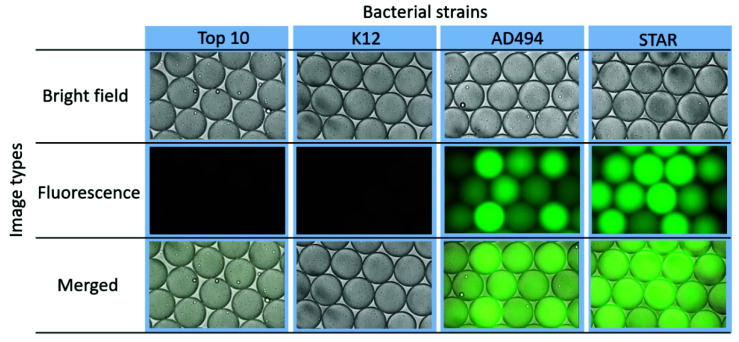
β-lactamase-producing bacteria showed fluorescence signals in microfluidic droplets.

Since each droplet can serve as an isolated bacterial culture, bacterial cells and the Fluorocillin sensor were co-encapsulated within microdroplets using a droplet-based microfluidic device, as shown in Figure 2. To ensure that the reaction verified in the bulk study can be repeated in droplets, bacterial cells were cultured in bulk with ampicillin-containing LB, and a relatively high number of bacterial cells (2 × 10^9^ bacterial cells per mL) were used for encapsulation within 50 µm in diameter droplets. Based on the calculation, the volume of the droplets is approximately 65 pL in volume, and 65 bacterial cells were encapsulated per droplet. The resistant strains AD494 and STAR showed strong fluorescence under the fluorescent microscope (Figure 7) and fluorescence intensity among different droplets varied, which may be caused by the deviations of bacterial numbers being captured by every individual droplet during droplet generation. The ampicillin-susceptible strains did not show any significant signals but only some weak background noise from the unreacted Fluorocillin.

**Figure 8:**
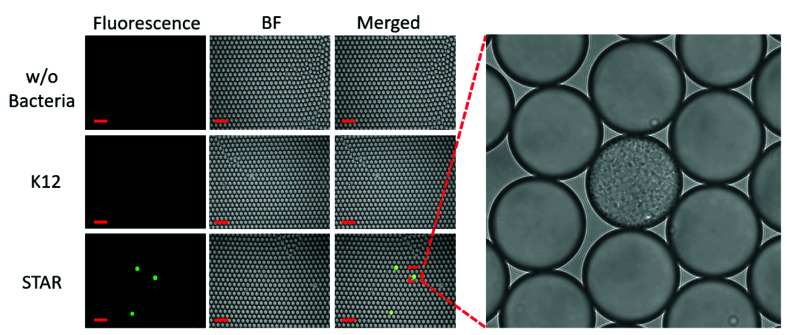
The single-cell culture within droplets to monitor ampicillin-resistant bacteria.

To determine if bacteria-containing droplets can be detected sooner by culturing in droplets instead of in a traditional liquid culture, we next encapsulated bacteria at the single-cell level in ampicillin-containing media along with the Fluorocillin sensor (Figure 8). Bacteria were cultured from frozen stock and then diluted to a level that ensures only a single bacterium was present in a positive droplet according to the Poisson distribution, such that droplets contained only one or zero bacteria [19]. Then, bacteria were cultured in droplets at 37 °C for 5 h and imaged by fluorescence microscopy. A single bacterial colony that developed from a single bacterium in a positive droplet was shown in the supplementary video file (S1_Droplet_Single_Colony.mp4).

A zoomed-in image shows that one of the positive droplets is filled with bacteria amplified from a single bacterium (Figure 8). Only the ampicillin-resistant bacteria strains grew in the droplets and reacted with the Fluorocillin sensor, resulting in a strong fluorescence signal after the 5-h culture. No fluorescent droplets were seen in the no-bacteria control and susceptible samples (K12). 

**Figure 9:**
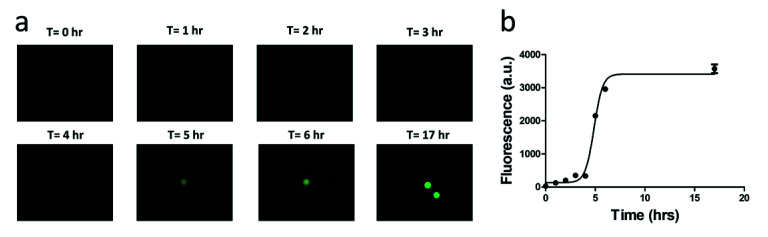
A time-course study of the fluorescent signal intensity of the single-bacterium droplets. (**a**) Microscopic images of the fluorescent droplet. (**b**) Fluorescence intensity of droplet.

In optimal conditions, *E. coli* divides roughly every 20 min, and so after 5 h, there are expected to be 32,768 bacteria formed from a single bacterium in a droplet. Signals are detectable under a fluorescent microscope after 5 h of culturing at 37 °C (Figure 9). The optical detector of the microscope is a CMOS (Complementary metal–oxide–semiconductor) camera array that converts the green fluorescent emission into a photocurrent. The PMT in the IC 3D system was more sensitive than the CMOS camera found in commercial microscopes. Therefore, we expected that a 5-h culture will give a strong enough fluorescent signal for detection by the IC 3D system.

The signal intensity of the single-bacterium droplets is a function of time of culturing (Figure 9). As bacteria numbers increase within a single droplet, the fluorescence signal rises until the Fluorocillin sensor is depleted. Detectable signals were observed after 5 h; however, the signal intensity increased by 6 h and saturated at 10 h of culturing. That said, 5 h of culture is sufficient for detection by the IC 3D system or a commercial fluorescent microscope.

### 3.3. Detection of Clinically Isolated Bacteria Using IC 3D System

**Figure 10:**
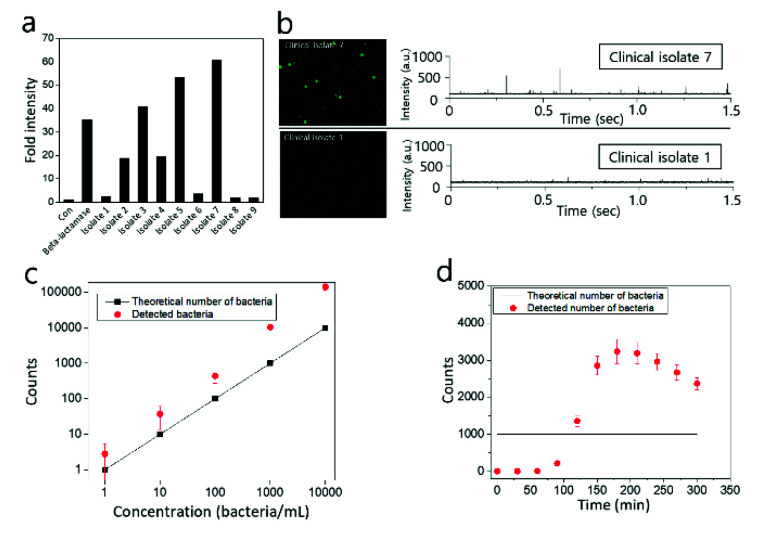
Detection of clinically isolated β-lactamase-producing bacteria with conventional well plate-based assay (**a**) or IC 3D (**b**). (**c**) Bacteria counts of β-lactamase-producing bacteria provided by the IC 3D fluorescence particle counter are compared with the theoretical number of bacteria. (**d**) Time-dependent measurement of 1000 STAR cells using IC 3D. The sample was encapsulated and analyzed every 30 min.

Finally, clinically isolated bacteria were identified with the Fluorocillin sensor using a conventional plate reader-based assay (Figure 10a) and IC 3D (Figure 10b). As shown in Figure 10a, ampicillin-resistant bacteria isolates (isolate 2, 3, 4, 5, and 7) were identified with bulk assay when 1 × 10^9^ bacterial cells were incubated with Fluorocillin for 30 min, and isolate 7 was considered to produce the highest amount of β-lactamase according to highest fluorescence intensity. Isolates 1, 6, 8, and 9 did not show significant fluorescence intensity, and commercial β-lactamase protein was used as a positive control to be compared to the β-lactamase-producing isolate groups.

Clinical isolates 1 and 7 were encapsulated within droplets (50 μm in diameter) with 2.5 μM Fluorocillin, and droplets were collected in a cuvette for the identification of β-lactamase-producing bacteria with IC 3D. After a 5 h incubation period at 30 °C, droplets were analyzed by a fluorescent microscope (Figure 10b, left panel) and the 3D particle counter (Figure 10b, right panel). Strong fluorescent droplets were observed from clinical isolate 7 (Figure 10b, upper panel), but there were no strong fluorescent droplets monitored with isolate 1 (Figure 10b, lower panel) when droplets were analyzed with a fluorescence microscope or 3D particle counter. The signal spikes shown in Figure 10b are voltage signals converted from fluorescence emissions by the PMT and the trans-impedance amplifier (TIA) in the IC 3D. The voltage spike profile in the time domain (Figure 1, left corner panel) was extracted and checked by the fitting software developed by Dr. Gratton (Laboratory for Fluorescence Dynamics, UC-Irvine, USA) [28,29]. The cuvette rotation speed and the sampling rate of the ADC are adjustable, but once the system was calibrated with the sample being used, these parameters were not changed during the experiments.

The counts from IC 3D matches with the plate count very well in the range of 100–10,000. Based on the data shown in Figure 10c, the detection limit of the IC 3D system is around 10 bacteria, which means 10 fluorescent droplets could be detected in 1 mL droplets. In this study, 1 mL samples containing 10 bacteria were converted into 60 μm droplets in diameter (approximately 110 pL in volume) using microfluidic device, which means there are 9 million droplets after microencapsulation and only 10 droplets contain a single bacterium each. In our previous report, it is possible to accurately quantify 5–10 fluorescent droplets [25,26], and a similar detection limit was achieved here for the monitoring of β-lactamase-producing bacteria. However, we also observed that the false-positive count was also increased when the number of bacteria was increased in a sample because the number of empty droplets have a higher chance of contacting bacteria-containing droplets, and β-lactamase can be transmitted into empty droplets thorough the contacting membrane between droplets. The other possibility can be the crosstalk between droplets by small broken droplets that can transfer β-lactamase into empty droplets, causing a false-positive count. To minimize the false-positive count, we also tested the effect of bacteria cultivation within droplets, and it was observed that the detected number of bacteria was higher than the theoretical number of bacteria when droplet-encapsulated bacteria were cultivated for more than 120 min (Figure 10d). This result suggests that 2-h incubation will be the optimum condition to minimize the false-positive count for the quantification of bacteria.

## 4. Discussion

In this study, β-lactamase-producing bacteria were detected using commercial Fluorocillin sensors in microfluidic droplets. The limit of detection was pushed to the single-digit level within less than 5 h. The comprehensive 3D particle counter was able to scan through the cuvette and report the positive counts in a few minutes, which reduces the sample-to-result turnaround time to less than 6 h. The single-bacterium detection in droplets took a longer time than the microplate bulk study due to the in-droplet culturing process. However, the detection sensitivity in droplets is 1000-fold stronger than the bulk reactor/detector. The sensors were commercially available, and the microfluidic chips are easy to make in a chemical fume hood. The 3D particle counter has been proved effective for detecting fluorescent particles, droplets, and cells rapidly. This study further demonstrated the capability of detecting a single bacterium from a low-concentration liquid sample.

Droplet generation was reproducible, and droplet stability was consistent throughout all the experiments. This was one of the benefits of using phenotypical droplet bacteria culture compared to droplet digital PCR thermal cycling, which may break a considerable number of droplets during thermocycling. Droplets were very stable at 37 °C. For milliliter-scale clinical samples, high-throughput microfluidic chips should be used not just for rapid encapsulation times, but also to avoid β-lactamase produced by the bacteria from reacting with the Fluorocillin in the syringe before they reach the nozzle of the microfluidic chips and are encapsulated in droplets, which may raise the background fluorescence of droplets that do not contain any bacteria.

Leaking is one of the weaknesses in droplet-based experiments because the sample concentration can be diluted, and it also causes contamination between droplets. It usually happens by droplet crosstalk because small molecules can be transmitted through the membrane barrier between droplets when droplets come into contact [30]. Broken tiny droplets also can transfer samples between droplets. In this study, a false-positive count was observed because of droplet crosstalk and broken droplets. To minimize this issue, droplet stability has to be guaranteed, and one potential approach can be the use of powerful surfactants that minimize the inter-droplet transfer of small molecules between droplets. 

Basically, an IC 3D system was designed to detect and quantify a small number of biological markers (nucleic acid, bacteria and cancer cells) from a relatively large volume of samples (100–1000 µL) [26]. Whole samples (1–2 mL) can be encapsulated within picoliter-sized droplets in 30 min for analyzing the converted droplets in 10 min. If the sample volume is less than 1 mL, it has to be diluted with a reaction buffer so they can provide similar background intensity to that of an empty droplet. This could be one of the weakness of the current IC 3D system, but this can be solved by reducing the analyzing cuvette size that has same height but a thinner width, so it can only contain a small amount of droplets (50–100 µL). 

Using microfluidic droplet cultures instead of microwells can reduce the reaction volume by more than 1,000,000 times and increase the effective concentration of cells for each reaction in a single picoliter-sized droplet. Therefore, the signal-to-background ratio from each reaction was boosted to a detectable level by the PMT in a short period. In this study, both engineered and clinical drug-resistant bacteria were used; however, there are many other β-lactamase-producing bacteria that were not tested in this study. In future studies, the Fluorocillin sensors should be tested with a wider spectrum of β-lactamases in both microwells and droplets and using different liquid biopsies, such as in blood or sputum, to further validate the effectiveness of the IC 3D technology.

## Supplementary Materials

The following are available online at https://www.mdpi.com/1424-8220/20/17/4667/s1, Video S1: Droplet Single Colony.
